# Supplementary material for: Gravitational scaling analysis on spatial diffusion of COVID-19 in Hubei Province, China
Source: PLoS One. 2021 Jun 11;16(6):e0252889. doi: 10.1371/journal.pone.0252889 (PMC8195435; doi:10.1371/journal.pone.0252889)
Supplement: S1 Table — The main results of multiple linear regression based on Eq (7) for local gravity modeling, including estimated parameter values and statistics, are listed in the table for reference and comparison. (DOCX) [file pone.0252889.s001.docx]

**S1 Table.** Parameters of gravity model and corresponding statistics of diffusion process of COVID-19 in Hubei Province

| Data | Parameters | | | Global statistics | | | Local statistics | | |
| --- | --- | --- | --- | --- | --- | --- | --- | --- | --- |
|  | Constant  term ln*η* | Size  exponent *υ* | Distance  exponent *β* | Goodness  of fit *R*^2^ | *F*-Statistic  *F* | coefficient of  variation *δ* | Probability  *P*_1_ | Probability  *P*_2_ | Probability  *P*_3_ |
| Jan. 27 | 0.1785 | 0.9807 | 0.3197 | 0.8746 | 48.8139 | 0.1442 | 0.8649 | 0.0000 | 0.0032 |
| Jan. 28 | 1.0188 | 0.8825 | 0.3042 | 0.8408 | 36.9654 | 0.1384 | 0.3624 | 0.0000 | 0.0062 |
| Jan. 29 | 1.3521 | 0.8659 | 0.2859 | 0.8334 | 35.0222 | 0.1281 | 0.2300 | 0.0000 | 0.0089 |
| Jan. 30 | 1.5516 | 0.8716 | 0.2676 | 0.8336 | 35.0594 | 0.1184 | 0.1663 | 0.0000 | 0.0120 |
| Jan. 31 | 1.5728 | 0.9013 | 0.2573 | 0.8923 | 57.9713 | 0.0894 | 0.0819 | 0.0000 | 0.0035 |
| Feb.1 | 1.6015 | 0.9375 | 0.2599 | 0.9008 | 63.5364 | 0.0847 | 0.0735 | 0.0000 | 0.0029 |
| Feb.2 | 1.4099 | 1.0018 | 0.2595 | 0.9120 | 72.5637 | 0.0810 | 0.1056 | 0.0000 | 0.0027 |
| Feb.3 | 1.7630 | 0.9751 | 0.2673 | 0.9052 | 66.8718 | 0.0808 | 0.0537 | 0.0000 | 0.0026 |
| Feb.4 | 1.8939 | 0.9952 | 0.2884 | 0.9137 | 74.0974 | 0.0775 | 0.0372 | 0.0000 | 0.0013 |
| Feb.5 | 1.9394 | 1.0171 | 0.2982 | 0.9151 | 75.4611 | 0.0772 | 0.0358 | 0.0000 | 0.0011 |
| Feb.6 | 2.0381 | 1.0240 | 0.3057 | 0.9222 | 83.0249 | 0.0732 | 0.0240 | 0.0000 | 0.0007 |
| Feb.7 | 2.1826 | 1.0270 | 0.3221 | 0.9248 | 86.0422 | 0.0721 | 0.0167 | 0.0000 | 0.0004 |
| Feb.8 | 2.1929 | 1.0389 | 0.3237 | 0.9241 | 85.2105 | 0.0724 | 0.0176 | 0.0000 | 0.0005 |
| Feb.9 | 2.3372 | 1.0344 | 0.3373 | 0.9222 | 83.0197 | 0.0733 | 0.0140 | 0.0000 | 0.0004 |
| Feb.10 | 2.4392 | 1.0321 | 0.3456 | 0.9251 | 86.4271 | 0.0716 | 0.0101 | 0.0000 | 0.0003 |
| Feb.11 | 2.4914 | 1.0337 | 0.3497 | 0.9232 | 84.1949 | 0.0724 | 0.0099 | 0.0000 | 0.0003 |
| Feb.12 | 2.8508 | 1.0380 | 0.4050 | 0.9247 | 85.9615 | 0.0739 | 0.0054 | 0.0000 | 0.0001 |
| Feb.13 | 2.9524 | 1.0388 | 0.4143 | 0.9354 | 101.3451 | 0.0680 | 0.0026 | 0.0000 | 0.0000 |
| Feb.14 | 3.0286 | 1.0365 | 0.4200 | 0.9393 | 108.2491 | 0.0656 | 0.0017 | 0.0000 | 0.0000 |
| Feb.15 | 3.1892 | 1.0195 | 0.4266 | 0.9492 | 130.7755 | 0.0591 | 0.0005 | 0.0000 | 0.0000 |
| Feb.16 | 3.2708 | 1.0135 | 0.4324 | 0.9507 | 134.9169 | 0.0580 | 0.0003 | 0.0000 | 0.0000 |
| Feb.17 | 3.3174 | 1.0117 | 0.4372 | 0.9500 | 132.8910 | 0.0585 | 0.0003 | 0.0000 | 0.0000 |
| Feb.18 | 3.3375 | 1.0121 | 0.4414 | 0.9497 | 132.1401 | 0.0589 | 0.0003 | 0.0000 | 0.0000 |
| Feb.19 | 3.3447 | 1.0132 | 0.4428 | 0.9490 | 130.2801 | 0.0594 | 0.0004 | 0.0000 | 0.0000 |
| Feb.20 | 3.3440 | 1.0142 | 0.4425 | 0.9493 | 131.0605 | 0.0592 | 0.0004 | 0.0000 | 0.0000 |
| Feb.21 | 3.4520 | 0.9976 | 0.4444 | 0.9499 | 132.6938 | 0.0582 | 0.0002 | 0.0000 | 0.0000 |
| Feb.22 | 3.4564 | 0.9982 | 0.4452 | 0.9501 | 133.3263 | 0.0581 | 0.0002 | 0.0000 | 0.0000 |
| Feb.23 | 3.4652 | 0.9980 | 0.4461 | 0.9503 | 133.9741 | 0.0580 | 0.0002 | 0.0000 | 0.0000 |
| Feb.24 | 3.4730 | 0.9980 | 0.4472 | 0.9505 | 134.3841 | 0.0579 | 0.0002 | 0.0000 | 0.0000 |
| Feb.25 | 3.4801 | 0.9978 | 0.4482 | 0.9506 | 134.6184 | 0.0579 | 0.0002 | 0.0000 | 0.0000 |
| Feb.26 | 3.4866 | 0.9978 | 0.4492 | 0.9507 | 134.8650 | 0.0579 | 0.0002 | 0.0000 | 0.0000 |
| Feb.27 | 3.4923 | 0.9976 | 0.4500 | 0.9508 | 135.3574 | 0.0578 | 0.0002 | 0.0000 | 0.0000 |
| Feb.28 | 3.5000 | 0.9973 | 0.4510 | 0.9508 | 135.2800 | 0.0578 | 0.0002 | 0.0000 | 0.0000 |
| Feb.29 | 3.5086 | 0.9971 | 0.4525 | 0.9509 | 135.6213 | 0.0578 | 0.0002 | 0.0000 | 0.0000 |
| Mar.1 | 3.5110 | 0.9971 | 0.4529 | 0.9510 | 135.7367 | 0.0578 | 0.0002 | 0.0000 | 0.0000 |
| Mar.2 | 3.5124 | 0.9972 | 0.4532 | 0.9510 | 135.8212 | 0.0578 | 0.0002 | 0.0000 | 0.0000 |
| Mar.3 | 3.5145 | 0.9971 | 0.4535 | 0.9510 | 135.8184 | 0.0578 | 0.0002 | 0.0000 | 0.0000 |

**Note:** The constant term is the logarithm of gravitational coefficient, while the size exponent and distance exponent are shown in the model. The coefficient of variation is the ratio of the standard error of regression to the average value of the logarithm of total number of confirmed cases. The three probability values represent the unacceptable levels of constant term, size exponent and distance exponent, which are equivalent to corresponding *t* statistics. The confidence level of the parameters = (1-probability value) * 100%.
