# Supplementary material for: Gravitational scaling analysis on spatial diffusion of COVID-19 in Hubei Province, China
Source: PLoS One. 2021 Jun 11;16(6):e0252889. doi: 10.1371/journal.pone.0252889 (PMC8195435; doi:10.1371/journal.pone.0252889)
Supplement: S2 Table — The main results of multiple linear regression based on Eq (8) for the local gravity modeling associated with spatial autoregressive process are listed in the table for reference and comparison. (DOCX) [file pone.0252889.s002.docx]

**S2 Table.** Gravity model parameters with spatial autoregressive terms and their corresponding probability values

| Date | Autoregressive gravity model parameters | | | | Probability value of regression coefficient | | | |
| --- | --- | --- | --- | --- | --- | --- | --- | --- |
|  | Gravitational coefficient | Size exponent | Distance exponent | Autoregressive coefficient | *P*_1_ | *P*_2_ | *P*_3_ | *P*_4_ |
| Jan. 27 | 0.2229 | 0.9837 | 0.3275 | -0.1125 | 0.8531 | 0.0000 | 0.0068 | 0.9354 |
| Jan. 28 | 1.1177 | 0.8848 | 0.3164 | -0.2110 | 0.3812 | 0.0001 | 0.0109 | 0.8755 |
| Jan. 29 | 1.3649 | 0.8678 | 0.2912 | -0.0031 | 0.2877 | 0.0001 | 0.0170 | 0.9980 |
| Jan. 30 | 1.5202 | 0.8740 | 0.2687 | 0.0679 | 0.2339 | 0.0001 | 0.0242 | 0.9544 |
| Jan. 31 | 1.4755 | 0.9030 | 0.2533 | 0.2066 | 0.1493 | 0.0000 | 0.0094 | 0.8192 |
| Feb.1 | 1.4222 | 0.9391 | 0.2494 | 0.3527 | 0.1581 | 0.0000 | 0.0095 | 0.6808 |
| Feb.2 | 1.2232 | 1.0040 | 0.2480 | 0.3419 | 0.2150 | 0.0000 | 0.0091 | 0.6771 |
| Feb.3 | 1.6933 | 0.9772 | 0.2655 | 0.1327 | 0.1023 | 0.0000 | 0.0071 | 0.8719 |
| Feb.4 | 1.7927 | 0.9970 | 0.2847 | 0.1957 | 0.0803 | 0.0000 | 0.0040 | 0.8037 |
| Feb.5 | 1.8427 | 1.0182 | 0.2954 | 0.2009 | 0.0761 | 0.0000 | 0.0034 | 0.7976 |
| Feb.6 | 1.9287 | 1.0251 | 0.3019 | 0.2195 | 0.0567 | 0.0000 | 0.0023 | 0.7686 |
| Feb.7 | 1.9910 | 1.0279 | 0.3119 | 0.3545 | 0.0493 | 0.0000 | 0.0017 | 0.6289 |
| Feb.8 | 1.9825 | 1.0399 | 0.3119 | 0.3770 | 0.0533 | 0.0000 | 0.0020 | 0.6095 |
| Feb.9 | 2.0853 | 1.0355 | 0.3222 | 0.4382 | 0.0474 | 0.0000 | 0.0018 | 0.5575 |
| Feb.10 | 2.1529 | 1.0332 | 0.3278 | 0.4903 | 0.0389 | 0.0000 | 0.0014 | 0.5026 |
| Feb.11 | 2.1754 | 1.0347 | 0.3294 | 0.5329 | 0.0400 | 0.0000 | 0.0015 | 0.4713 |
| Feb.12 | 2.5455 | 1.0395 | 0.3863 | 0.5064 | 0.0238 | 0.0000 | 0.0006 | 0.5027 |
| Feb.13 | 2.6314 | 1.0405 | 0.3943 | 0.5222 | 0.0139 | 0.0000 | 0.0003 | 0.4549 |
| Feb.14 | 2.6938 | 1.0381 | 0.3989 | 0.5426 | 0.0103 | 0.0000 | 0.0002 | 0.4220 |
| Feb.15 | 2.8157 | 1.0199 | 0.4029 | 0.6178 | 0.0039 | 0.0000 | 0.0001 | 0.3087 |
| Feb.16 | 2.8802 | 1.0135 | 0.4075 | 0.6485 | 0.0029 | 0.0000 | 0.0000 | 0.2762 |
| Feb.17 | 2.9152 | 1.0116 | 0.4114 | 0.6661 | 0.0028 | 0.0000 | 0.0000 | 0.2672 |
| Feb.18 | 2.9450 | 1.0120 | 0.4165 | 0.6523 | 0.0028 | 0.0000 | 0.0000 | 0.2799 |
| Feb.19 | 2.9462 | 1.0130 | 0.4175 | 0.6627 | 0.0029 | 0.0000 | 0.0000 | 0.2754 |
| Feb.20 | 2.9463 | 1.0140 | 0.4173 | 0.6611 | 0.0029 | 0.0000 | 0.0000 | 0.2749 |
| Feb.21 | 3.0698 | 0.9974 | 0.4205 | 0.6379 | 0.0019 | 0.0000 | 0.0000 | 0.2847 |
| Feb.22 | 3.0772 | 0.9980 | 0.4215 | 0.6333 | 0.0019 | 0.0000 | 0.0000 | 0.2873 |
| Feb.23 | 3.0867 | 0.9978 | 0.4225 | 0.6326 | 0.0018 | 0.0000 | 0.0000 | 0.2867 |
| Feb.24 | 3.0965 | 0.9978 | 0.4238 | 0.6297 | 0.0018 | 0.0000 | 0.0000 | 0.2885 |
| Feb.25 | 3.1043 | 0.9976 | 0.4249 | 0.6289 | 0.0017 | 0.0000 | 0.0000 | 0.2889 |
| Feb.26 | 3.1120 | 0.9976 | 0.4260 | 0.6272 | 0.0017 | 0.0000 | 0.0000 | 0.2899 |
| Feb.27 | 3.1193 | 0.9974 | 0.4269 | 0.6249 | 0.0016 | 0.0000 | 0.0000 | 0.2910 |
| Feb.28 | 3.1298 | 0.9971 | 0.4282 | 0.6204 | 0.0016 | 0.0000 | 0.0000 | 0.2950 |
| Feb.29 | 3.1411 | 0.9970 | 0.4299 | 0.6160 | 0.0016 | 0.0000 | 0.0000 | 0.2985 |
| Mar.1 | 3.1446 | 0.9970 | 0.4304 | 0.6142 | 0.0016 | 0.0000 | 0.0000 | 0.2999 |
| Mar.2 | 3.1463 | 0.9970 | 0.4307 | 0.6137 | 0.0016 | 0.0000 | 0.0000 | 0.3002 |
| Mar.3 | 3.1487 | 0.9969 | 0.4311 | 0.6133 | 0.0016 | 0.0000 | 0.0000 | 0.3006 |
