# Supplementary material for: Gravitational scaling analysis on spatial diffusion of COVID-19 in Hubei Province, China
Source: PLoS One. 2021 Jun 11;16(6):e0252889. doi: 10.1371/journal.pone.0252889 (PMC8195435; doi:10.1371/journal.pone.0252889)
Supplement: Attachment — Submitted filename: Comments to PONE-D-21-07713.docx [file pone.0252889.s004.docx]

Comments to the Author

The authors used gravitational scaling analysis to study the spatial transmission of COVID-19 in Hubei, China. I provided my feedbacks based on my first reading and my experiences.

Abstract:

The manuscript needs to explore the results more deeply. I don’t think the main result in the Abstract “The local gravity model based on power law decay can effectively describe the diffusion patterns and process of COVID-19 in Hubei Province, and the goodness of fit of the gravity model based on negative exponential decay to the observational data is not satisfactory” is informative, or useful. The authors only described the model function, not the COVID-19 transmission. Most readers are not interested in the goodness of fit or confidence level, which should be within the acceptable level, as default.

Conclusion part in the abstract is more important, which should replace the current results part. Yet, the authors need to clarify some information here: what does long-range effect mean, please explain? “the size of a city and the distance …affect the total number of confirmed cases”, positively affect or negatively affect, or just affect? What are direct and horizontal transmission?

The authors need definitely rewrite the abstract. Please refer to some journals’ guidance, like landscape ecology, whose abstract is structured.

Main text:

Since 2020, there have been a series of COVID-19 papers published, many of which focus on spatial transmission, like epidemiology study. The authors need to make a brief review/comparison of these publications, since the authors’ stance is from the urban geography perspective, your strengthen?

Section 3.1 should not place in the Results part.

Table 2. I cannot directly find useful information from this long table. Suggestion, plot some columns?

The authors’ work focuses on Wuhan, the first city attacked by the COVID-19 as reported. It would be useful to make a comparison study, with other provinces (sub-nation unit) in China or other countries using the model.

OVERALL

The paper provides an important perspective in studying COVID-19 transmission, which is useful and has potential implications in policy making. I hope that my feedback comments are useful to help improve it in this regard.
