# Supplementary material for: Gravitational scaling analysis on spatial diffusion of COVID-19 in Hubei Province, China
Source: PLoS One. 2021 Jun 11;16(6):e0252889. doi: 10.1371/journal.pone.0252889 (PMC8195435; doi:10.1371/journal.pone.0252889)
Supplement: Attachment — Submitted filename: Response to Reviewers - 2021-04-21.docx [file pone.0252889.s005.docx]

# Response to Reviewers

### Reviewer #1:

It is a good paper with a clear presentation of the model, its implementation in modeling the pandemic, and a thorough interpretation of the results.

My comments are minor and discretionary:

**[Reviewer #1]** 1. The distance decay functions employed by the study include power and exponential functions. I understand that the two are the most popular ones used in the literature. The literature also suggests additional functions such as square-root exponential and log-normal (Taylor, 1983, Distance decay in spatial interactions. In Concepts and Techniques in Modern Geography) and Gaussian (Shi et al. 2012, Annals of AAG 102, 1125-1134). Future work may expand the selection set.

**[Replay]** Thank you for your advice. I know there are many forms of distance decay functions (Table A1). A distance decay function is actually associated with a probability density function, and the basis of probability distribution functions are the principle of entropy maximization (Table A2). However, according to the spatial characteristics of Gaussian distribution, the normal function is not suitable for the impedance function of a gravity models.

**Table A1** General typology of distance decay functions

| Name | Function | Linear regression transformation |
| --- | --- | --- |
| General Model: | $T=ae^{-bf(d)}$ |  |
| Single Log Models: |  |  |
| Normal | $T_{ij}=ae^{-bd_{ij}^{2}}$ | $\ln T_{ij}=\ln a-bd_{ij}^{2}$ |
| Exponential | $T_{ij}=ae^{-bd_{ij}}$ | $\ln T_{ij}=\ln a-bd_{ij}$ |
| Square root exponential | $T_{ij}=ae^{-bd_{ij}^{0.5}}$ | $\ln T_{ij}=\ln a-bd_{ij}^{0.5}$ |
| Double Log Models: |  |  |
| Pareto model | $T_{ij}=ae^{-b\ln d_{ij}}$ | $\ln T_{ij}=\ln a-b\ln d_{ij}$ |
| Lognormal | $T_{ij}=ae^{-b(\ln d_{ij})^{2}}$ | $\ln T_{ij}=\ln a-b(\ln d_{ij})^{2}$ |
| Notation: *T_ij_*=Interaction between locations *i* and *j*. *d_ij_*=Distance between *i* and *j*. *a*, *b*=Constants. *e*=Exponential constant (2.7183). | | |

**Source**: (1) Taylor PJ (1975). Distance decay models in spatial interaction. Norwich, pp.23-24. (2) Haggett, *et al*, 1977, pp.31; (3) Zhou (1995), Page 360.

**Table A2** Three probability distribution functions based on entropy-maximization principle

| **Interval** | **Probability density function** | **Characteristic scale** | **Maximum entropy** |
| --- | --- | --- | --- |
| $a\leq x\leq b$ | $f(x)=\frac{1}{b-a}$ | Range: *b*-*a* | $\ln(b-a)$ |
| $0\leq x<\infty$ | $f(x)=\frac{1}{\mu}\exp(-\frac{x}{\mu})$ | Mean value: *μ* | $\ln(\mu e)$ |
| $-\infty<x<\infty$ | $f(x)=\frac{1}{\sqrt{2\pi}\sigma}\exp(-\frac{x^{2}}{2\sigma^{2}})$ | Standard error: *σ* | $\ln(\sqrt{2\pi}\sigma e)$ |

**Note**: This table is summarized by reference to Reza (1961, page 268) and Silviu (1977, page 298). As for the parameters, *μ* refers to average value, *σ* to standard error, and *e*, the base of the natural system of logarithms, having a numerical value of approximately 2.7183.

Now, a paragraph is added to the Discussion section to clarify the reasons of choosing impedance functions as follows:

The gravity models are based on distance decay law which can be expressed by a certain type of impedance function. In geography, the impedance functions are also termed distance decay functions, which are not limited to negative exponential function and inverse power function. General typology of distance decay functions include power function, exponential function, square root exponential function, normal function, lognormal function, gamma function, and so on (Batty and Longley, 1994; Haggett *et al*, 1977; Shi *et al*, 2012; Taylor, 1975; Taylor, 1983; Zhou, 1995). In fact, linear function and logarithmic function can also serve as distance decay functions. These distance decay functions fall into two categories: one is those that can be directly derived from the principle of entropy maximization, including linear decay function, exponential function, normal distance, and power function; the other is those that cannot be directly derived by means of the entropy-maximizing method, including logarithmic function, lognormal function, square root exponential function, and gamma function (Table 4). Among various distance decay functions, the inverse power function is special. On the one hand, it can be derived from double entropy maximization processes, and on the other, it has no characteristic scale (Chen, 2008). In this work, the main ideas of choosing distance decay functions are as follows. *First, the principle of spatial entropy maximization*. Geographical gravity and spatial interaction modeling are associated with the process of maximizing entropy (Batty, 2010; Chen, 2015; Wilson, 1968; Wilson, 1970; Wilson, 2010). In this sense, only linear function, exponential function, power function and normal function are acceptable for gravity modeling. *Second, the notion of spatial complexity*. Geographical systems are complex spatial systems (Allen, 1997; Batty, 2005; Portugali, 2011; Wilson, 2000). Generally speaking, complex systems bear no characteristic scales and do not satisfy linear spatial process (Chen, 2008). Therefore, linear functions can be excluded. *Third, spatial diffusion characteristics*. Distance decay results in special spatial distribution states. Spatial distribution and probability distribution are essentially two different aspects of the same problem. There are three probability density distribution functions which are associated with the principle of entropy maximization. That is, uniform distribution (linear function), negative exponential distribution (exponential function), and normal distribution (Gaussian function) (Chen, 2008). If the distance variable *r* has a clear lower limit *a* and an upper limit *b* (i.e., -*a*<*r*<*b*, and *a*≥0, *b*>0 represent constants), the probability density based on entropy maximization satisfies the uniform distribution and can be described by a linear function; If the distance variable *r* has a clear lower limit *r*=0, but no upper limit (i.e., 0≤*r*<∞), the entropy-maximization-based probability density meets the exponential distribution and can be modeled by an exponential function; If the distance variable *r* has neither a lower limit nor an upper limit (i.e., -∞<*r*<∞), the probability density based on entropy maximization takes on the normal distribution (Silviu, 1977; Reze, 1961). Spatial diffusion starts from a certain center and spreads to the periphery, which belongs to the second case mentioned above. A pair of exponential decay processes compose a power law decay process (Chen, 2008). So, the normal function can be excluded in this work, despite the fact that it is useful in geographical spatial analysis (Shi *et al*, 2012). Finally, only negative exponential function and inverse power law function can be utilized in the local gravity modeling.

**Table 4** The common distance decay functions in geographical analyses and its mathematical and physical properties

| Type | Name | Function | Entropy maximization | Characteristic scale |
| --- | --- | --- | --- | --- |
| Linear | Linear Model: | $T(r)=T_{0}-kr$ | Yes | Yes |
| Single logarithm | Normal | $T(r)=T_{0}\exp(-kr^{2})$ | Yes | Yes |
|  | Exponential | $T(r)=T_{0}\exp(-kr)$ | No | Yes |
|  | Square root exponential | $T(r)=T_{0}\exp(-kr^{1/2})$ | No | Yes |
|  | Logarithmic | $T(r)=T_{0}-k\ln r$ | No | Yes |
| Double logarithms | Power law | $T(r)=T_{1}r^{-\alpha}$ | Yes | No (scaling) |
|  | Lognormal | $T(r)=T_{1}\exp(-k(\ln r)^{2})$ | No | Yes |
| Mixed | Gamma | $T(r)=T_{1}r^{-\alpha}\exp(-kr)$ | No | Yes |

**Notes**: (1) Notation: *T*=Interaction between two locations; *r*=Distance between two locations; *T*_0_, *T*_1_, *k*, *α*=Constants; *e*=Exponential constant (2.7183). (2) References: Batty and Longley, 1994; Haggett *et al*, 1977; Taylor, 1975; Zhou, 1995. (3) Properties: the relationships between the distance decay functions and entropy maximization and characteristic scale are summarized in this study.

- Allen PM (1997). *Cities and Regions as Self-Organizing Systems: Models of Complexity*. Amsterdam: Gordon and Breach Science Pub.
- Batty M (2005). *Cities and Complexity: Understanding Cities with Cellular Automata, Agent-Based Models, and Fractals*. Cambridge, MA/London, England: The MIT Press
- Batty M, Longley PA (1994). *Fractal Cities: A Geometry of Form and Function*. London: Academic Press
- Chen YG (2008). *Fractal Urban Systems: Scaling, Symmetry, and Spatial Complexity*. Beijing: Science Press [In Chinese]
- Portugali J (2011). *Complexity, Cognition and the City*. Berlin: Springer
- Reza FM (1961). *An Introduction to Information Theory*. New York: McGraw Hill
- Shi X, Alford-Teaster J, Onega T, Wang D (2012). Spatial access and local demand for major cancer care facilities in the United States. *Annals of the Association of American Geographers*, 102(5): 1125-1134
- Silviu G (1977). *Information Theory with Applications*. New York: McGraw Hill
- Taylor PJ (1975). Distance decay models in spatial interaction. *Norwich*, pp.23-24
- Wilson AG (2000). *Complex Spatial Systems: The Modelling Foundations of Urban and Regional Analysis*. Singapore: Pearson Education Asia Pte Ltd.

**[Reviewer #1]** 2. On interpretation/discussion of the result, I am not surprised by the strong effect of Wuhan on peripheral cities and no much between peripheral cities themselves, which is consistent with the spatial interaction pattern in passenger flows (even before the pandemic) in Hubei. In other words, if actual passenger flows could be obtained in the region (which is not easy in China), one could predict/explain the pandemic by that observed data without modeling the interaction.

**[Replay]** I quite agree with you. In a companion paper of this paper, we study the correlation between the numbers of confirmed cases of COVID-19 and passenger flows of all provinces, municipalities, and autonomous regions in Chinese mainland. Your judgment is absolutely correct. However, this kind of research cannot reveal the mathematical law behind the spatial diffusion of COVID-19.

Novel coronavirus pneumonia is not the real objective of the article. The real purpose of this article is not to study the spatial spread of Hubei's new crown pneumonia. The real purpose of this paper is to validate a local gravity model by means of the data sets of novel coronavirus pneumonia. There is evidence that new technology diffusion also meets this model.

**[Reviewer #1]** 3. I like the information presented in Table 2 and Figure 2, which are considered the main results of the paper. However, Table 2 seems to be redundant as Figure 2 tells the same info with better visual effect.

**[Replay]** Now, Table 2 has been removed from the text.

**[Reviewer #1]** 4. The lockdown in late Jan in the region completely brought population movement to a stop. I'd suspect that the temporal lag in the diagnosis cases in various cities reflects the incubation period.

**[Replay]** It's possible, but there's no evidence. This is interesting. We plan to research this problem.

### Reviewer #2:

**[Reviewer #2]** This research proposes an integrated mathematical model combining gravity model with spatial autoregression model to investigate the spatial diffusion of COVID-19 in Hubei province, China. Generally, this manuscript provides us sufficient information about model selection and establishment, data source, and statistical test. The effectiveness of the proposed method was tested empirically and statistically. The analytical results are of certain enlightening value for understanding the spatial diffusion process of COVID-19, especially in terms of the effects of population size and spatial distance. However, to be accepted for publication, the presentation still need improvement. Detailed comments are as follows:

**[Reviewer #2]** The mathematical model proposed in this study is suitable for certain conditions. Specifically, the gravity model describes the one-directional transmission of COVID-19 from Wuhan to other cities, and the spatial autoregression model considers horizontal diffusion between cities (except Wuhan) within Hubei province. More details about the epidemic prevention and control measures (i.e., spatial isolation of cities and the corresponding time spots of the implementation) should be provided to illustrate that the practical situation in Hubei is consistent with applicable conditions of the model.

**[Replay]** Thanks. Two paragraphs have been added to the text to explain the applicable conditions of the model.

“In this study, the allometric scaling relation between passenger flow and number of confirmed cases can be treated as basic postulate for mathematical derivation.”

“The main task of sciences is to make models (Neumann, 1961), and models reflect the quintessential human activity (Holland, 1998). The basic role of models is to explain and predict (Batty, 1991; Kac, 1969; Fotheringham and O’Kelly, 1989). However, models are not universally applicable. All mathematical models have their own scope of application. The principal applicable conditions of the local gravity models can be summarized as follows. (1) Generally, the models can be applied to the single center diffusion process based on core-periphery interaction. (2) The relation between the number of confirmed cases and passenger flow follow allometric scaling law. (3) The model based on negative exponential decay is suitable for the spatial process with locality (simpler spatial systems with characteristic scale), while the model based on inverse power law decay is suitable for the spatial process of long-range action (complex spatial systems without characteristic scale).”

- Batty M (1991). Cities as fractals: Simulating growth and form.In: Crilly AJ, Earnshaw RA, Jones H (eds). *Fractals and Chaos*. New York: Springer–Verlag, pp43–69
- Fotheringham AS, O'Kelly ME (1989). *Spatial Interaction Models: Formulations and Applications*. Boston: Kluwer Academic Publishers
- Holland JH (1998). *Emergence: from Chaos to Order*. Cambridge, Massachusetts: Perseus Books
- Kac M (1969). Some mathematical models in science. *Science*, 166: 695-699
- Neumann J von (1961). *Collected works (Vol.6)*．New York/Oxford: Pergamon Press

### **Reviewer #3: Comments to the Author**

**[Reviewer #2]** The authors used gravitational scaling analysis to study the spatial transmission of COVID-19 in Hubei, China. I provided my feedbacks based on my first reading and my experiences.

**[Reviewer #2]** Abstract: The manuscript needs to explore the results more deeply. I don’t think the main result in the Abstract “The local gravity model based on power law decay can effectively describe the diffusion patterns and process of COVID-19 in Hubei Province, and the goodness of fit of the gravity model based on negative exponential decay to the observational data is not satisfactory” is informative, or useful. The authors only described the model function, not the COVID-19 transmission. Most readers are not interested in the goodness of fit or confidence level, which should be within the acceptable level, as default.

**[Replay]** This paper belongs to the quantitative studies, which differ significantly from qualitative studies where methods are concerned. The former is based on data analysis to verify or falsify a model, while the latter is based on data analysis to make interpretation of meaning of observational facts (Table C1). Further, the quantitative studies fall into two categories: theoretical research and empirical research (Table C2). The former is to make theoretical models by means of postulate-modeling process, while the latter is to sort out observational data based on the idea of hypothesis-testing process. As John von Neumann (1961) pointed out: “The sciences (for theoretical studies) do not try to explain, they hardly even try to interpret, they mainly make (mathematical) models.” I am very much in favor of his viewpoint of Louf and Barthelemy (2014), who said: “The success of natural sciences lies in their great emphasis on the role of quantifiable data and their interplay with models. Data and models are both necessary for the progress of our understanding: data generate stylized facts and put constraints on models. Models on the other hand are essential to comprehend the processes at play and how the system works. If either is missing, our understanding and explanation of a phenomenon are questionable. This issue is very general, and affects all scientific domains, including the study of cities.”

**Table C1 Traditional distinctions that are associated with quantitative and qualitative methods**

**Table C2 A comparison between theoretical research and empirical research based on quantitative analysis**

| Type | Theoretical research | Empirical research |
| --- | --- | --- |
| Mathematical tools | Make models | Processing data |
| Basis of method | Mathematics | Statistics |
| Method | Postulate-modeling | Hypothesis-testing |
| Aim | Developing theories | Solving practical problems |

A scientific research includes only two processes (or elements): one is description (how), and the other is understanding (why). “They (scientists) understood that science should proceed first by describing how things work and later by understanding why.”(Gordon, 2005, page 293) Description is based on measurements and mathematics, and understanding is based on investigation, experiments, simulation, and computation. “The development and establishment of what is usually taken to be the characteristic methodology of science has always been regarded as constructive of the Scientific Revolution. The two main elements of this scientific method are the use of mathematics and measurement to give precise determinations of how the world and its parts work, and the use of observation, experience, and where necessary, artificially constructed experiments, to gain understanding of nature.” (Henry, 2002, page 14)

- Gordon K (2005). The mysteries of mass. *Scientific American*, 293(1): 40-46/48
- Henry J (2002). *The Scientific Revolution and the Origins of Modern Science (2nd Edition)*. New York: Palgrave
- Louf R, Barthelemy M (2014). Scaling: lost in the smog. *Environment and Planning B: Planning and Design*, 41: 767-769
- Neumann J von (1961). Collected works (Vol.6). New York/Oxford: Pergamon Press, 492

**[Reviewer #2]** Conclusion part in the abstract is more important, which should replace the current results part. Yet, the authors need to clarify some information here: what does long-range effect mean, please explain? “the size of a city and the distance …affect the total number of confirmed cases”, positively affect or negatively affect, or just affect? What are direct and horizontal transmission?

**[Replay]**

Q1: what does long-range effect mean, please explain?

**[Reply 1]** In fact, the long-range effect and locality have been explained in the text.

“The mathematical properties of two decay functions are different. The exponential function bears a parameter indicating characteristic scale, while the power function has no parameter representing characteristic scale. The former implies simplicity, while the latter implies complexity (Barabasi and Bonabeau, 2003). As for the negative exponential decay, the spatial autocorrelation function of is a tailing-out curve, while the partial autocorrelation function is a cutoff-tailed curve. It implies that negative exponential decay essentially reflects a local action which lacks direct long range effect, and therefore it is inconsistent with the first law of geography. Both the autocorrelation function and partial autocorrelation function of inverse power law decay are tailing off, so it indicates the long range effect, which is consistent with the first law of geography.”

**Table 2 Comparison of goodness of fit of gravity models based on different distance decay functions**

| Distance function | Autocorrelation | Partial autocorrelation | Space effect | Epidemic diffusion in Hubei Province |
| --- | --- | --- | --- | --- |
| Negative exponential function | Tailed | Truncated | Quasi Locality: simple, limited scope, inconsistent with the first law of geography | Poor fitting effect |
| Negative power function | Tailed | Tailed | Long range effect: complex, unlimited scope, consistent with the first law of geography | Good fitting effect |

What is more, I have published several papers to explain the long-range effect and locality in geographical processes.

**Figure 5 The schematic histograms of spatial ACF and PACF based on an inverse power function and a negative exponential function**

**Note**: (1) For the negative exponential decay, the spatial autocorrelation function (ACF) of is a tailing-out curve, while the partial autocorrelation function (PACF) is a cutoff-tailed curve. (2) Both the autocorrelation function (ACF) and partial autocorrelation function (PACF) of inverse power law decay are tailing off, so it indicates the long range effect, which is consistent with the first law of geography.

Q2: “the size of a city and the distance …affect the total number of confirmed cases”, positively affect or negatively affect, or just affect?

**[Reply 2]** To my thinking, the formula has shown the positively influence and negatively influence.

$N_{j}=\eta P_{j}^{\upsilon}r_{j}^{-\beta}$. (5)

For the size measure, the scaling exponent is positive, indicating positive influence, while the distance exponent is negative, showing negative influence.

What is more, in the Introduction, there is a sentence as follows: “Generally speaking, spatial diffusion is directly proportional to size and inversely proportional to distance, which reflects how gravity take effects in geographical systems.”

Now, two sentences are added to the end of equation (5) to explain the properties of influence:

“where *η*>0 is the rescaled gravity coefficient, *β*>0 is the rescaled distance exponent, and *υ*>0 is the rescaled size calibration parameter. Since both *β* and *υ* are greater than 0, the positive sign (+) means a positive influence of city sizes, and the negative sign (-) means a negative influence of distance.”

Q3: What are direct and horizontal transmission?

**[Reply 3]** A paragraph was added to the footnotes in Figure 1 as below:

“The line segments illustrate the vertical diffusion from Wuhan to other cities in Hubei. The horizontal diffusion relationship between other cities outside Wuhan are not shown. The vertical diffusion is described by local gravity model, and the horizontal diffusion is tested by spatial autoregressive analysis.”

**[Reviewer #2]** The authors need definitely rewrite the abstract. Please refer to some journals’ guidance, like landscape ecology, whose abstract is structured.

**[Replay]** As a matter of fact, I write the abstract with reference to the following textbooks about scientific papers:

- Bjorn Gustavii (2002). *How to Write and Illustrate a Scientific Paper*. Cambridge: Cambridge University Press
- Robert A. Day, Barbara Gastel (2003). *How to Write and Publish a Scientific Paper (Sixth Edition)*. Cambridge: Cambridge University Press

For example, according to “How to Write and Publish a Scientific Paper” (edited by Robert A. Day and Barbara Gastel), the Abstract (of a paper) should include: (1) **[Objectives and scope]** state the principal objectives and scope of the investigation, **(2) [Method]** describe the methods employed**, (3) [Result]** summarize the results, and **[Conclusions],** state the principal conclusions.

**[Reviewer #2]** Main text: Since 2020, there have been a series of COVID-19 papers published, many of which focus on spatial transmission, like epidemiology study. The authors need to make a brief review/comparison of these publications, since the authors’ stance is from the urban geography perspective, your strengthen?

**[Replay]** A series of COVID-19 papers published, but they are different in property from this paper. In fact, I have reviewed four papers about COVID-19 recently.

About the Components of the Discussion, Robert A. Day and Barbara Gastel (2003) pointed out: “What are the essential features of a good Discussion? The main components will be provided if the following injunctions are heeded. 1. Try to present the principles, relationships, and generalizations shown by Results. And bear *in mind, in a good Discussion, you discuss—you do not recapitulate—the Results*. 2. Point out any exceptions or any lack of correlation and define unsettled points. Never take the high-risk alternative of trying to cover up or fudge data that do not quite fit. **3. Show how your results and interpretations agree (or contrast) with previously published work.** 4. Don’t be shy; discuss the theoretical implications of your work, as well as any possible practical applications. 5. State your conclusions as clearly as possible. 6. Summarize your evidence for *each* conclusion. Or, as the wise old scientist will tell you, ‘Never assume anything except a 4% mortgage.’” Bjorn Gustavi (2002)’s book entitled "how to write and illustrate a scientific paper" has a similar view.

Several sentences have been added to the Discussion section as below:

“A series of COVID-19 papers published have been published, showing many interesting research results. However, in terms of research objectives, results and nature, this paper is quite different from the published papers. The application of mathematical tools in scientific research has two main functions: one is to sort out observational data (hypothesis-testing process), and the other is make theoretical models (postulate-modeling process). In the previous works about COVID-19, mathematics was mainly employed to process data, while this paper is to develop theoretical model of spatial diffusion.”

**Table A3 The similarity and differences between this paper and the papers on COVID-19 written by other researchers**

| Mathematical method function | Field | Method | Example |
| --- | --- | --- | --- |
| Building theoretical models | Mathematical geography | Postulate-modeling | This paper |
| Sorting out observational data | Statistical geography | Hypothesis-testing | A series of COVID-19 papers published |

- Bjorn Gustavii (2002). *How to Write and Illustrate a Scientific Paper*. Cambridge: Cambridge University Press
- Robert A. Day, Barbara Gastel (2003). *How to Write and Publish a Scientific Paper (Sixth Edition)*. Cambridge: Cambridge University Press

**[Reviewer #2]** Section 3.1 should not place in the Results part.

**[Replay]** Subsection 3.1 has been revised as subsection 2.4.

**[Reviewer #2]** Table 2. I cannot directly find useful information from this long table. Suggestion, plot some columns?

**[Replay]** Now, according to the suggestion of Reviewer #1, table 2 has been changed into one of a Supporting Information files.

In fact, table 2 shows the changing trend of model parameters and the corresponding statistics (F statistics, coefficient of variation). The statistics can be used to evaluate the model parameters, and the model parameters reflects the micro-level geographical information.

In Subsection 3.2 (now, Subsection 3.1), I pointed out: “The analytical process of geographical mathematical modeling involves three basic aspects. The first is mathematical structure, the second is model parameters, and the third is statistics such as correlation coefficient and standard errors.”

**[Reviewer #2]** The authors’ work focuses on Wuhan, the first city attacked by the COVID-19 as reported. It would be useful to make a comparison study, with other provinces (sub-nation unit) in China or other countries using the model.

**[Replay]** This is one of the series papers. A companion paper of this paper was devoted to exploring the spatial diffusion of COVID-19 in the whole China.

This paper is devoted to deriving a local gravity model to describe the spatial diffusion process based on core-periphery relations. In other provinces (sub-nation units), no clear core-periphery diffusion patterns can be found.

Now, a conjecture is added to the Discussion section as below:

“In a sense, this paper is devoted to developing a local gravity model to describe the spatial diffusing processes based on core-periphery patterns. Maybe the model can be applied to core-periphery diffusion process in other fields such as information technology diffusion. The diffusion of the movable type printing press is an interesting phenomenon (Arbesman, 2012; Dittmar, 2011). Likely, this technology diffusion process and patterns can be characterized by the local gravity model.”

- Arbesman S (2012). *The Half-Life of Facts: Why Everything We Know Has An Expiration Date*. New York: Penguin Group
- Dittmar JE (2011). Information technology and economic change: The impact of the printing press. *The Quarterly Journal of Economics*, 126(3): 1133–1172

**[Reviewer #2]** OVERALL: The paper provides an important perspective in studying COVID-19 transmission, which is useful and has potential implications in policy making. I hope that my feedback comments are useful to help improve it in this regard.

**[Replay]** I try my best to revise this paper by referring to your comments. Thanks.
